# Why super sandstorm 2021 in North China?

**DOI:** 10.1093/nsr/nwab165

**Published:** 2021-09-02

**Authors:** Zhicong Yin, Yu Wan, Yijia Zhang, Huijun Wang

**Affiliations:** Key Laboratory of Meteorological Disaster, Ministry of Education/Joint International Research Laboratory of Climate and Environment Change (ILCEC)/Collaborative Innovation Center on Forecast and Evaluation of Meteorological Disasters (CIC-FEMD), Nanjing University of Information Science and Technology, Nanjing 210044, China; Southern Marine Science and Engineering Guangdong Laboratory (Zhuhai), Zhuhai 519080, China; Nansen-Zhu International Research Centre, Institute of Atmospheric Physics, Chinese Academy of Sciences, Beijing 100029, China; Key Laboratory of Meteorological Disaster, Ministry of Education/Joint International Research Laboratory of Climate and Environment Change (ILCEC)/Collaborative Innovation Center on Forecast and Evaluation of Meteorological Disasters (CIC-FEMD), Nanjing University of Information Science and Technology, Nanjing 210044, China; Key Laboratory of Meteorological Disaster, Ministry of Education/Joint International Research Laboratory of Climate and Environment Change (ILCEC)/Collaborative Innovation Center on Forecast and Evaluation of Meteorological Disasters (CIC-FEMD), Nanjing University of Information Science and Technology, Nanjing 210044, China; Key Laboratory of Meteorological Disaster, Ministry of Education/Joint International Research Laboratory of Climate and Environment Change (ILCEC)/Collaborative Innovation Center on Forecast and Evaluation of Meteorological Disasters (CIC-FEMD), Nanjing University of Information Science and Technology, Nanjing 210044, China; Southern Marine Science and Engineering Guangdong Laboratory (Zhuhai), Zhuhai 519080, China; Nansen-Zhu International Research Centre, Institute of Atmospheric Physics, Chinese Academy of Sciences, Beijing 100029, China

**Keywords:** sandstorm, dust source, Arctic sea ice, La Niña, cyclone

## Abstract

Severe sandstorms reoccurred in the spring of 2021 after an absence for more than 10 years in North China. The dust source area, located in Mongolia, suffered destructive cooling and warming in early and late winter, which loosened the land. A lack of precipitation, excessive snow melt and strong evaporation resulted in dry soil and exiguous spring vegetation. A super-strong Mongolian cyclone developed on the bare and loose ground, and easily blew and transported large amounts of sand particles into North China. Furthermore, top-ranking anomalies (sea ice shift in the Barents and Kara Sea, and sea surface temperatures in the east Pacific and northwest Atlantic) were found to induce the aforementioned tremendous climate anomalies in the dust source area. Analyses, based on large-ensemble Coupled Model Intercomparison Project Phase 6, yield results identical to the reanalysis data. Thus, the climate variabilities at different latitudes and synoptic disturbances jointly facilitated the strongest spring sandstorm over the last decade.

## Teaser

Large climate anomalies and strong weather disturbance jointly contributed to super sandstorm 2021 in North China that reoccurred after absence for more than 10 years.

## INTRODUCTION

Sandstorms (visibility <1 km) are natural phenomena characterized by strong winds and blowing sand particles, and frequently occur in arid and semi-arid regions. Strong sandstorms can result in loss of topsoil and productivity in the dust source area, and damage human health and agriculture along the dust path. Dust aerosols can also influence both weather and climate through radiative and cloud-related processes [1,2]. Strong gusts are closely related to mid-latitude cyclone activities on both a synoptic and interannual-decadal timescale [3–5]. Severe synoptic cyclones destroy the land surface, dynamically lift and transport sand particles downstream, and finally deposit grit on the ground (e.g. North China). In spring, cyclogenesis frequently occurs over Mongolia and the north of China, providing dynamic conditions for the development of dust [3,4,6]. Moreover, the persistent dry climate and loose land surface in these regions are indispensable background conditions for dust weather. That is, Mongolia and the north of China are important dust source areas for sandstorms in North China [6,7].

Numerous large-scale climate factors were revealed to influence the frequency of spring dust weather in North China. Kang and Wang [8] identified a positive correlation between the number of dust days and the intensity of the East Asian winter monsoon (EAWM), and found that a weak winter monsoon could not induce dust weather in North China. The negative phase of the Arctic Oscillation enhanced atmospheric instability, making north–south movement of weather disturbances easier, and consequently increased the frequency of spring dust [9]. The Antarctic Oscillation is one of the dominant patterns of tropospheric circulation in the southern hemisphere, and plays an important role in variations of dust weather frequency in North China via the meridional teleconnection from the Antarctic to the Arctic [10]. In 2000 and 2001, a La Niña pattern developed, which significantly enhanced the EAWM and could have contributed to the destructive dust weather in North China [11–13]. In terms of Arctic sea ice, its relationship with spring dust weather exhibited decadal changes (i.e. significant during 1996–2014 but insignificant prior to 1996). Decreased sea ice in the Barents Sea stimulated a stationary Rossby wave train, which increased the cyclogenesis and atmospheric thermal instability in North China [14]. Notably, most previous studies have focused on the impacts of climate variability on cyclogenesis and its associated strong gusts, but little is known about the impacts on large anomalies in dust sources.

**Figure 1.**
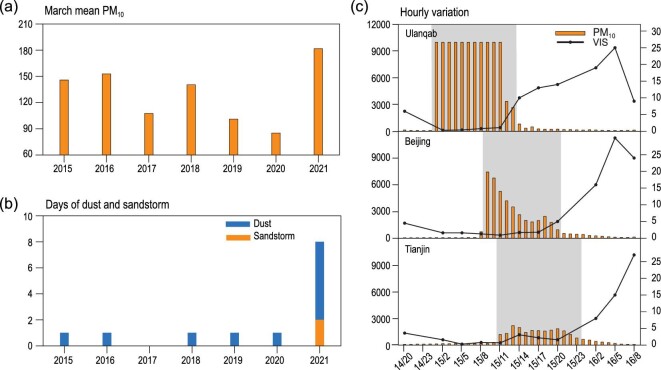
Observations of sandstorms in North China. (a) March mean PM_10_ concentrations (unit: μg m^−3^) in North China (34–42°N, 105–120°E) from 2015 to 2021. (b) Number of March dust (blue) and sandstorm (orange) days in Beijing from 2015 to 2021. (c) Hourly PM_10_ concentrations (orange bars) and 3-hour observed visibility (unit: km, black lines) along the dust path (Ulanqab, Beijing and Tianjin) from 14 to 16 March 2021. The gray shadow indicates the period with high PM_10_ concentrations (the upper monitoring threshold is 9985 μg m^−3^).

The frequency of spring dust weather was high from the 1950s to 1970s, but low from the 1980s to 1990s [8]. In the 21st century, the number of dust days has been persistently low but with two remarkable peaks in 2000–2002 and in ∼2006 [14]. In the last decade, no dust storm has occurred in March in North China. The March mean PM_10_ concentration in North China reached its highest in 2021 (181.8 μg m^−3^) and was 39% larger than the mean of 2015–2020 (Fig. 1a). This is because severe dust weather occurred between 14–16 (strong sandstorm), 19–21 (blowing sand) and 27–29 (strong sandstorm) March 2021 (Fig. S1a). For example, only 0–1 day with dust occurred in Beijing from 2015 to 2020, but the number of dust days sharply increased to 8 (with 2 sandstorm days) in March 2021 (Fig. 1b). These two sandstorm processes detrimentally influenced 3.8 and 2.7 million square kilometers, respectively. Between 14–16 March 2021, the PM_10_ concentration exceeded the monitoring threshold in Ulanqab (>9985 μg m^−3^) and reached extraordinarily high values in Beijing (>7400 μg m^−3^) and Tianjin (>2200 μg m^−3^) along the dust path (Fig. S1b). These ‘reoccurred’ strong sandstorms in 2021, after an absence of sandstorms for more than 10 years, have carried huge implications and received great attention. However, to the best of our knowledge, the climate-synoptic reasons are still unknown. In this comprehensive study, we aim to emphasize the sub-seasonal variability of the dust source area, to analyze the effects of cyclonic circulation (taking the sandstorm on 15 March 2021 as an example) and to further reveal the joint forces from preceding climate factors.

## LARGE CLIMATE ANOMALIES IN DUST SOURCE AREA

**Figure 2.**
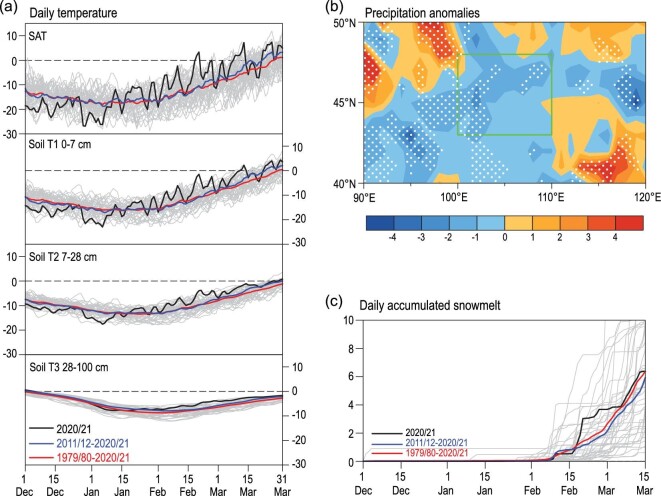
Climate conditions of the dust source area. (a) Daily variation in surface air temperature and soil temperature (unit: °C. Underground depth: 0–7 cm, 2–28 cm and 28–100 cm) from 1 December to 31 March in 2020/21 (black), 2011/12–2020/21 mean (blue), 1979/80–2020/21 mean (red) and each year during 1979/80–2019/20 (gray). (b) The precipitation anomalies (unit: 10^–3^ mm) from 1 December 2020 to 15 March 2021 relative to the mean of 2011/12–2020/21. The white dots indicate that the anomalies in 2020/21 exceeded the 85th percentile of anomalies from 2011/12 to 2020/21. The green box represents the dust source area. (c) Daily variation in accumulated snowmelt (unit: 10^–2^ mm) in the dust source area from 1 December to 15 March in 2020/21 (black), 2011/12–2020/21 mean (blue), 1979/80–2020/21 mean (red) and each year during 1979/80–2019/20 ( gray).

**Figure 3.**
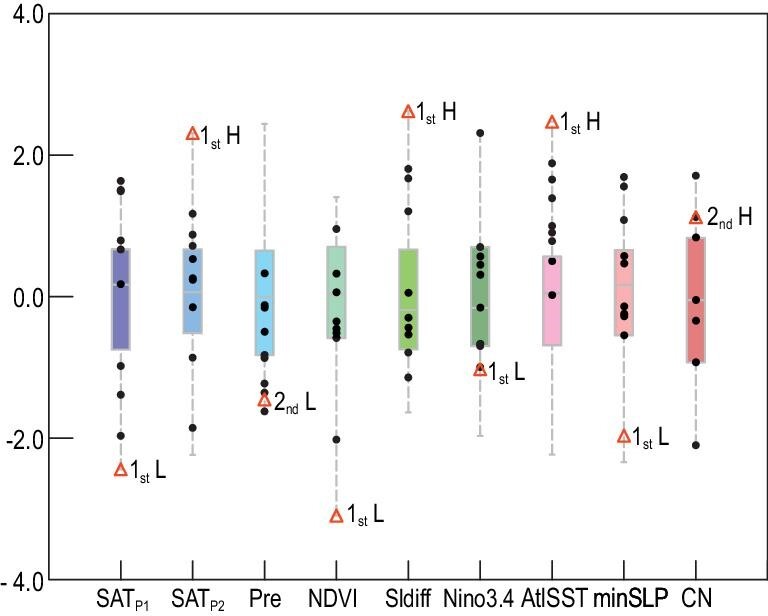
Normalized factors influencing North China dust weather during 1979/80–2020/21 (boxplot), 2011/12–2019/20 (black dots) and in 2020/21 (red triangles). The climate conditions of the dust source area include surface air temperature during P1 (SAT_P1_) and P2 (SAT_P2_), 1 December–15 March precipitation (Pre) and FM NDVI. The preceding factors that influenced dust source include difference of sea ice in Barents and Kara Sea (SIdiff, JF minus ND), SON Nino3.4 index and NDJ Northwest Atlantic SST (AtlSST). The atmospheric indexes include minimum March SLP (minSLP) and March cyclone numbers (CN) over the dust source area. The high (H) and low (L) ranks of these factors within 2011/12–2020/21 are also marked.

At 07 : 00 BJT on 15 March 2021, the PM_10_ concentration in Beijing began to increase, indicating that sandstorm weather had come into the capital of China (Fig. 1c). Using the Hybrid Single-Particle Lagrangian Integrated Trajectory (HYSPLIT) model [15], the 24-hour backward trajectory of the sand particles was calculated, revealing that the dust source area was located in Mongolia (Fig. S1b). An upstream, loose and shattered land surface is a necessary material basis of dust weather. Thus, we analyzed the climate conditions in Mongolia from December 2020 to March 2021. During December and the first half of January (P1), the surface air temperature (SAT) in the dust source area was persistently lower than the mean of 2011/12–2020/21, and was accompanied by lower underground soil temperatures up to 28 cm deep (Fig. 2a, S3). In contrast, the surface air and underground soil (even in the 28–100 cm soil layer) became continuously warmer than the climate mean from 16 January to 15 March (P2). Quantitatively, in P1 and P2, the SAT anomalies were –3.9°C and +4.1°C with respect to the mean of 2011/12–2020/21 in the dust source area, respectively (Fig. S2). Furthermore, the rank of these two SAT anomalies during P1 (SAT_P1_) and P2 (SAT_P2_) in 1979/80–2020/21 were the first low and first high, indicating a dramatic reversal of thermal conditions (Fig. 3). Previous studies have clearly shown that a preceding colder SAT is conducive to the occurrence of spring dust weather, as cold thermal conditions result in a deeper and more frozen soil zone. When strong warming occurred in the dust source area, the land surface evidently loosened and severe desertification came subsequently [4,10]. According to the shift in SAT and soil temperature, the soil in Mongolia was significantly frozen during P1 but was sharply thawed during P2, and thus, the conditions of the land surface became conducive to dust storms before 15 March 2021. Similar SAT shifts have been detected in other years with frequent dust (e.g. 1980/81 and 2000/01 in Fig. S4). Furthermore, the date of permafrost melting (defined as 0°C in 0–7 cm soil [16]) was ∼10 days earlier than the climate mean (Fig. 2a, S3), which also provided favorable conditions for the earlier appearance of sandstorms in 2021.

Dry soil was another factor that enhanced the dust source. No effective precipitation occurred in the entire period from 1 December 2020 to 15 March 2021, which resulted in negative precipitation anomalies in Mongolia (Fig. 2b). The rank of the precipitation amount was the second (third) smallest during 2011/12–2020/21 (1979/80–2020/21), indicating the severe drought in Mongolia (Fig. 3). The high SAT also indicated a strong ground surface evaporation, which accelerated land drying. Although snowmelt resulting from atmospheric heating alleviated the drought in the dust source area to some extent, it could not overcome the joint impacts of deficient precipitation and strong surface evaporation (Fig. S5). The accumulated snowmelt significantly exceeded the climate mean (Fig. 2c) and thus further caused the ground to become bare and loose. Miao *et al*. [17] found that the Normalized Difference Vegetation Index (NDVI) in Mongolia was positively correlated with precipitation and was negatively correlated with SAT. Significant anomalies in winter precipitation and the SAT_P2_ in 2021 resulted in the worst vegetation cover during 1979/80–2020/21 (Fig. 3). Accordingly, sparse vegetation obviously contributed to the occurrence of the sandstorm in the spring of 2021 [18].

## IMPACTS OF ATMOSPHERIC CIRCULATIONS AND PRECEDING FORCINGS

**Figure 4.**
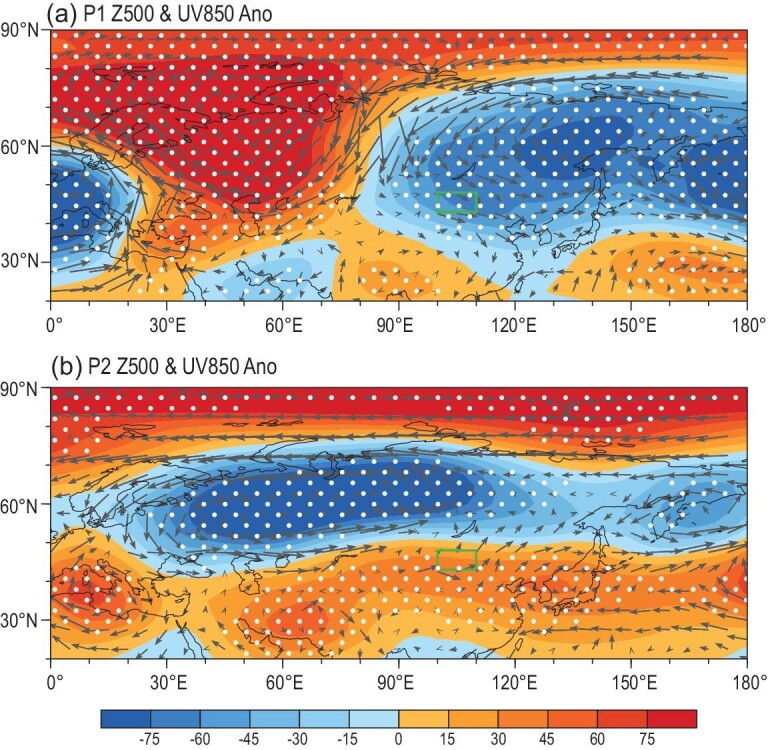
Anomalies of 500 hPa geopotential height (unit: gpm, shading) and 850 hPa wind (unit: m s^–1^, vector) during (a) P1 and (b) P2 relative to the mean of 2011/12–2020/21. The white dots indicate that the anomalies in 2020/21 exceeded the 85th percentile of anomalies from 2011/12 to 2020/21. The green box represents the dust source area.

Large-scale atmospheric circulations not only influenced local precipitation and SAT, but also bridged the preceding climate forcings that contained predictive and multi-layered interacting information. During P1 in 2021, there were large positive anomalies at 500 hPa geopotential height over the Ural Mountains and negative anomalies over the Okhotsk Sea, indicating a stronger Ural blocking high and a deeper East Asian deep trough (Fig. 4a). This intensified EAWM extremely guided the cold air mass from the polar region to south of 30°N, resulting in a lower SAT [19] and less precipitation in the mid-high latitude of East Asia [20,21]. Evident sub-seasonal changes also existed in anomalous atmospheric circulations. The anomalies at 500 hPa geopotential height shifted to a zonal distribution during P2, in which there were positive anomalies over the polar and subtropical regions and negative anomalies over the mid-latitudes (Fig. 4b). Under such large-scale atmospheric backgrounds, the cold air mass was trapped over the West Siberian Plain and the East European Plain. The above-normal atmospheric high, located over the east of China and Mongolia, caused increased SAT in Mongolia and the east of China during P2 (Fig. S2b). Precipitation was also sparse because the cold air and warm air diverged prior to arriving in Mongolia (Fig. 4b).

**Figure 5.**
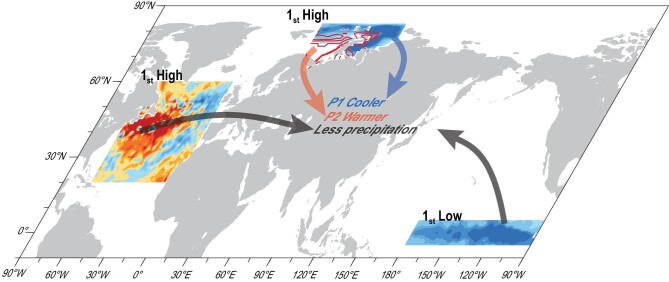
Schematic diagram of how preceding forcings (ranks during 2011/2012–2020/21 are labeled) influenced the climate anomalies in the dust source area in 2020/21. Negative anomalies of ND BKSI (shading) resulted in lower SAT_P1_ in Mongolia, while positive JF BKSI anomalies (red contours) led to higher SAT_P2_ in 2021. Coolest SON east Pacific and warmest NDJ northwest Atlantic during 2011/12–2020/21 jointly contributed to less winter precipitation in Mongolia.

Changes in the Barents and Kara Sea ice (BKSI) have been recognized as an effective factor driving the atmospheric anomalies that influence the winter–spring Eurasian climate. The loss of BKSI weakened the meridional temperature gradient and westerly component, initiated an increase in the local geopotential height [22] and then enhanced the Ural blocking high in the mid-troposphere and the Siberia high near the surface [23]. The accompanying anomalous northerlies could transport Arctic air mass to cause a persistently cooling climate in Siberia and the Mongolian Plateau [24] (Fig. 4a). The singular value decomposition (SVD) between the observational November–December (ND) sea ice and the Eurasian SAT_P1_ during 1979/80–2020/21 reproduced the revealed relationship, i.e. decreased ND BKSI resulted in lower SAT_P1_ (Fig. S6a and b). Within a large ensemble of the Coupled Model Intercomparison Project Phase 6 (CMIP6), identical SVD results were also obtained, indicating robust causal connections (Fig. S6c and d). The ND BKSI in 2020 was observed to have evident negative anomalies and was a preceding signal of the lower SAT_P1_ (Fig. 5). However, the January–February (JF) BKSI anomalies in 2021 changed to largely positive (Fig. 5) and were speculated to be responsible for the higher SAT_P2_. To verify this speculation, SVD analysis was also applied to the JF BKSI and SAT_P2_ using both reanalysis data and CMIP6 historical simulations. These SVD results also supported the theory that the increased JF BKSI led to higher SAT_P2_ (Fig. S7). In addition, the anomalous atmospheric circulations associated with BKSI anomalies were composited for P1 and P2 (Fig. S8a and c), and were similar to the observed anomalies in 2020/21 (Fig. 4) and significantly resulted in the SAT changes (Fig. S8b and d). The reversal of BKSI was evaluated as the JF BKSI minus the ND BKSI. This index in the winter of 2020/21 was the highest during 1979/80–2020/21 (Fig. 3), and likely contributed to the sharp shift in SAT in Mongolia (Fig. 2a).

In addition to large BKSI anomalies, there were also evident negative anomalies of September–November (SON) sea surface temperature (SST) in the east Pacific (i.e. La Niña) and positive November–January (NDJ) SST anomalies in the northwest Atlantic (NWAtlSST) in 2020/21 (Fig. 5). The thermal conditions of the sea surface imply a memory effect and air–sea interactions that could significantly impact atmospheric circulations in the following months and seasons. The Atlantic Multidecadal Oscillation (AMO) has exhibited a positive phase since the mid-1990s [25]. Geng *et al*. [26] showed that La Niña coincided with the strengthened EAWM during a positive AMO phase. Influenced by strong EAWM, the water vapor flux easily diverged around Mongolia [27] and precipitation consequently decreased [28]. Similarly, lower mean winter precipitation in the dust source area followed the La Niña event by means of SVD (Fig. S9a and b). The P1 and P2 mean precipitation were separately used in the SVD analysis, and a similar relationship was reproduced (figure not shown). Large-ensemble CMIP6 simulations also suggested that the La Niña event in the tropical Pacific resulted in drought in Mongolia in winter to a large extent (Fig. S9c and d). The atmospheric anomalies associated with cooler SST in the east Pacific were also composited (Fig. S10a) and were conducive to less precipitation (Fig. S10b). Accordingly, the strongest La Niña during 2011/12–2020/21, occurring in 2020 (Nino3.4 index = –1.3°C), played an important role in the moisture deficit in the dust source area (Fig. 3).

The warmer NWAtlSST and associated non-adiabatic heating was remotely linked to positive height anomalies over the Urals by an upper Rossby wave-like train across the North Atlantic and coastal Europe, which was detected in both the atmospheric general circulation model [29] and observations [30]. Moreover, when the positive anomalies of the SON NWAtlSST persisted into December and January, the warmer sea surface could efficiently heat the air column above, which finally resulted in a weak tropospheric Asian polar vortex in winter [31]. Both the strengthened Ural High and the weakened Asian polar vortex were closely related to the decrease in winter precipitation in Mongolia [32]. The SVD results, based both on long-term reanalysis and CMIP6 data, successfully verified the negative correlation between winter (as well as P1 and P2) precipitation in Mongolia and the NDJ NWAtlSST (Fig. S11). The atmospheric anomalies associated with warmer NWAtlSST were also composited (Fig. S10c) and were conducive to less precipitation (Fig. S10d). In NDJ 2020/21, the NWAtlSST was the warmest (+1.2°C) during 1979/80–2020/21 (Figs 3 and 5), and contributed to the drought in Mongolia. Overall, the reversal of the BKSI anomalies, the La Niña event and the warmer northwest Atlantic in 2020/2021 (Fig. 3) jointly contributed to a loose and dry land surface, generating sufficient dust sources in Mongolia (Fig. 2).

## SYNOPTIC LIFTING AND TRANSPORTATION OF SANDS

As aforementioned, persistent and extreme climate anomalies in the winter of 2020/21 provided sufficient dust sources in Mongolia. Another necessary triggering mechanism was the strong cyclonic winds and thermal instability that lifted the sand from the bare and loose land surface and transported it to North China and the surrounding area. Referring to the calculation approach of Fu *et al*. [33], the frequency of Mongolian cyclones in March 2021 was the second-highest between 2011/12 and 2020/21. The lowest sea level pressure (SLP) inside could indicate the intensity of the cyclones. The strongest (third strong) Mongolian cyclone during 2011/12–2020/21 (1979/80–2020/21) happened on 14–15 March 2021 (Fig. 3), effectively triggering the strongest sandstorm in the last decade.

**Figure 6.**
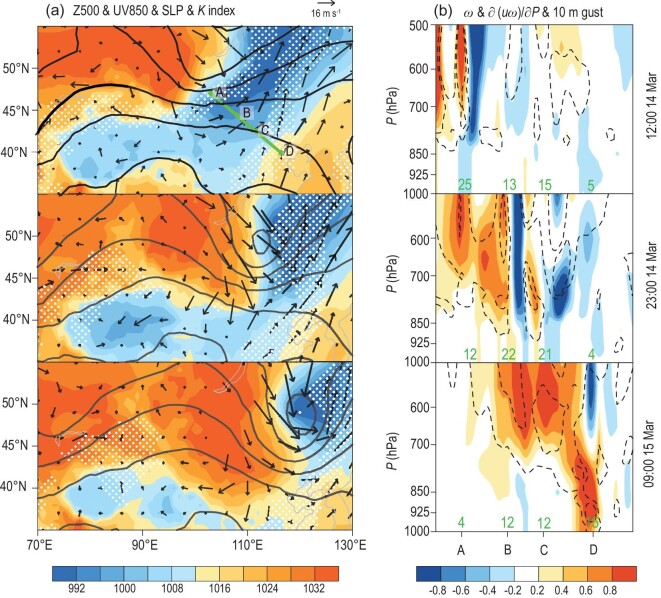
Atmospheric circulations associated with sands lifting and transportation. (a) 500 hPa geopotential height (unit: gpm, contours), 850 hPa wind (unit: m s^–1^, vector), sea level pressure (unit: hPa, shading) and *K* index (>0, white dots) at 12 : 00, 23 : 00 on 14 March 2021 and 09 : 00 on 15 March 2021 Beijing Time (BJT). (b) The profile along the dust path (green line in panel (a)) including vertical velocity (unit: Pa s^–1^, shading) and the vertical transport of westerly momentum (unit: 10^–4^ m s^–2^, <0, dashed contours). The green numbers at the bottom represent the 10m gust speed (unit: m s^–1^).

In Fig. 6a, line A–D illustrates the dust path simulated by the HYSPLIT model (Fig. S1b), and Points A, B, C and D are located near the center of the dust source area, northern border of inner Mongolia, Ulanqab and Beijing, respectively. At 12 : 00 on 14 March 2021, a strong Mongolian cyclone (min SLP = 984 hPa) occurred both near the surface and in the lower and mid-troposphere. The 500 hPa trough line was backward-tilting (relative to the SLP), indicating that this Mongolian cyclone was unstable (*K* index > 0) and would powerfully develop in the following 1–2 days (Fig. 6a). The descending motions with downward transport of westerly momentum (i.e. ∂(*uω*)/∂*P*) dramatically enlarged the northerly (10 m gust = 25 m s^–1^), which shook and blew the dry and loose land surface. Subsequently, the ascending motions in front of the Mongolian cyclone immediately lifted the sand particles into the troposphere (Fig. 6b). With time, the Mongolian cyclone strengthened (min SLP = 978 hPa), and the strong northerly wind moved southwards to Points B (10 m gust = 22 m s^–1^) and C (10 m gust = 21 m s^–1^) at 23 : 00 on 14 March 2021 (Fig. 6b). At 09:00 on 15 March 2021, the cold advection reached Beijing, carrying large amounts of sand and dust particles. The tropospheric westerly momentums were transported downward to the surface, resulting in large gusts (15 m s^–1^). Together with the evident instability, severe sandstorms (visibility < 0.5 km, PM_10_ > 7400 μg m^−3^ and duration ≈ 16 hours) happened in Beijing and the surrounding area (Fig. 1c).

## CONCLUSION AND DISCUSSION

As revealed by many previous studies [14,34], there were a small number of dust days in North China after 2006 and sandstorms have been absent for 10–20 years in Beijing prior to 2021. In autumn and winter of 2020/2021, several top-ranking climate anomalies in the Arctic sea ice, Pacific and Atlantic SST were observed, which were supposed to helpfully induce the strongest spring sandstorm in North China. The greatest reversal of the sea ice anomalies in the Barents and Kara Sea (i.e. negative anomalies in November and December changed to positive anomalies in January and February) resulted in a sharp change in the SAT and underground soil temperature from cooler in early winter to warmer in late winter. Consequently, the evident heating to a deeper frozen soil level easily led to a loose and bare land surface. In addition, the coolest sea surface in east Pacific (La Niña) and warmest sea surface in northwest Atlantic during 2011/12–2020/21 jointly contributed to less precipitation in Mongolia, thus enhancing the dust sources with high air temperature in late winter. Due to changes in SAT and precipitation, the intensified melting of permafrost and snow and the poor spring vegetation cover further caused the ground to be bare and loose, providing favorable dust sources for the earlier occurrence of sandstorms in the spring of 2021. The super-strong Mongolian cyclone (min SLP = 978 hPa) developed on 14–15 March 2021 and triggered the strongest March sandstorm in the last decade. The northerly advection, downward transport of westerly momentum and strong instability resulted in strong gusts (∼20 m s^–1^) that shook, blew, lifted and transported large amounts of sand and dust particles into North China. In Beijing, visibility decreased to <500 m and the PM_10_ concentrations increased to >7400 μg m^−3^. Overall, the joint effects of climate variabilities at different latitudes (preceding climate forcing in Fig. S12), atmospheric responses (large-scale atmospheric anomalies in Fig. S12), anomalous dust sources and synoptic disturbances (direct causes in Fig. S12) led to the occurrence of the strongest spring sandstorm in North China over the last decade.

In addition to the dust weather on 14–15 March, several dust events also occurred in March–May 2021. The SAT in Mongolia was still higher than the climate mean in April and May (Fig. S13a). Although effective precipitation happened in late April, it was not sufficient to change the dry status of the soil moisture (Fig. S13a). Under such meteorological conditions, the land surface was still covered with sparse vegetation, and the bare and loose land unceasingly provided dust sources. Although we did not analyze the connections between cyclogenesis and preceding climate variability, such information has been documented in many previous studies. For example, negative anomalies of BKSI (Fig. 5) favored decreased snow cover over western Siberia and have enhanced the cyclogenesis since the mid-1990s [14]. The occurrence of La Niña (Fig. 5) and warmer tropical western Atlantic (Fig. S14) favored the upper trough over regions of East Asian cyclogenesis in mid-high latitudes [35]. Therefore, strong gusts frequently occurred in the dust source area from March to May (Fig. S13b), and most of the gusty winds from Mongolia corresponded to obvious increases in the PM_10_ concentration in Beijing (Fig. S13c). In this study, we synthetically employed site observations, reanalysis data, HYSPLIT tracing and CMIP6 historical simulations to explain the climate-synoptic inducements of the strongest sandstorm of the last decade. However, simulations by numerical dust model are strongly required to understand the relative contributions of climate variability, climate change and land mismanagement in further research.

## DATA AND METHODS

### Observations of dust weather

PM_10_ concentrations have been widely observed since 2015 in China, and are publicly available at https://www.aqistudy.cn/historydata/. The upper monitoring threshold of the PM_10_ concentration is 9985 μg m^−3^. The number of dust and sandstorm days in Beijing and 3 h observed visibility were obtained from the National Meteorological Information Center of the China Meteorological Administration.

### ERA5 reanalysis data

The fifth-generation European Center for Medium Range Weather Forecasts [36] provided acknowledged daily and monthly reanalysis data of the atmosphere and underlying surface from 1979 to the present. The atmospheric data (1° × 1°) included the geopotential at 500 hPa, wind at 850 hPa, sea level pressure, SAT, evaporation, soil temperature, soil moisture, snowmelt, precipitation, relative humidity, vertical velocity, 10 m gusts and *K* index. The vertical transport of westerly momentum was defined as ∂(*uω*)/∂*P* [37], where ∂(*uω*)/∂*P* < 0 represents downward transport. In addition, the monthly sea ice concentration and SST data had a 1° × 1° resolution.

### GLDAS data

The evaporation and soil temperature data were downloaded from a dataset of the Global Land Data Assimilation System (https://ldas.gsfc.nasa.gov/data) [38].

### Vegetation cover

The NDVI quantifies the vegetation by measuring the difference between near-infrared and red light. Gridded NDVI data from 1979/80 to 2020/21 were obtained from the National Oceanic and Atmospheric Administration's (NOAA) National Centers for Environmental Information [39].

### CMIP6 historical simulations

To verify the causal relationship between the preceding forcing factors and the climate conditions in the dust source area, the CMIP6 data were used to repeat the statistical analysis. Daily and monthly data from 1979 to 2014 in the historical simulations were employed in this study [40], including precipitation, surface air temperature, sea ice concentrations and SST. For uniformity, we chose 35 models that contained all of the variables we used.

### HYSPLIT trajectories

We employed the HYSPLIT model to track the back trajectory of the sandstorm that occurred on 15 March 2021 in Beijing and determine the dust source area. This model calculation is a hybrid of the Lagrangian approach and Eulerian methodology [15]. In addition to code execution, HYSPLIT can be run interactively on the website: https://www.ready.noaa.gov/index.php.

## Supplementary Material

nwab165_Supplemental_File
