# Supplementary material for: Why super sandstorm 2021 in North China?
Source: Natl Sci Rev. 2021 Sep 2;9(3):nwab165. doi: 10.1093/nsr/nwab165 (PMC8900684; doi:10.1093/nsr/nwab165)
Supplement: nwab165_Supplemental_File [file nwab165_supplemental_file.pdf]

Supplementary

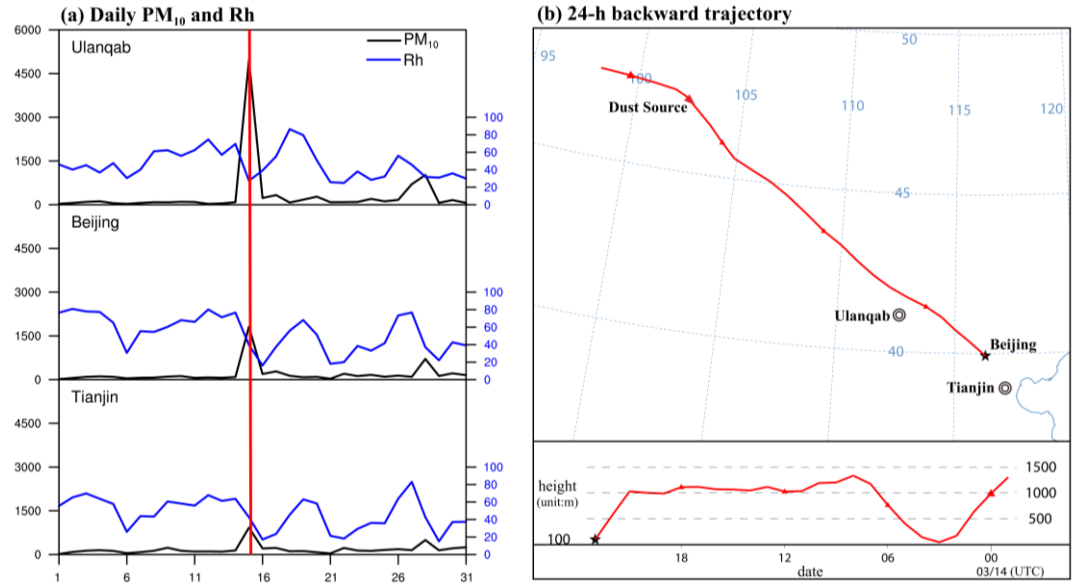

Figure S1. (a) Daily variation in PM_{10} concentrations (unit: μg m^{-3}, black) and relative humidity (unit: %, blue) along the dust path (i.e., Ulanqab, Beijing and Tianjin) in March 2021. The red line represents the date of 15 March 2021. (b) The 24-hour backward trajectory of the sandstorm with terminus in Beijing at 15 March 2021 07:00 BJT. This trajectory is based on the HYSPLIT model (https://www.ready.noaa.gov/hypub-bin/trajsrc.pl) and its height-time profile is illustrated by the red line of the lower part of panel b. The horizontal axis and the vertical axis in the bottom represents the date and the height (unit: m), respectively.

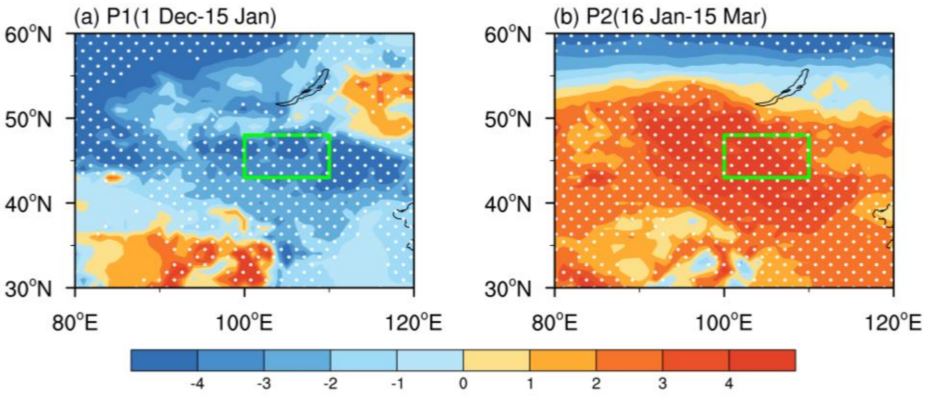

Figure S2. The surface air temperature annomalies (unit: °C) during 1 December 2020 to 15 January 2021 (P1) and 16 January 2021 to 15 March 2021 (P2) relative to the mean of 2011/12-2020/21. The white dots indicate that the anomalies in 2020/21 exceeded the 85th percentile of anomalies from 2011/12 to 2020/21. The green box represents the dust source area.

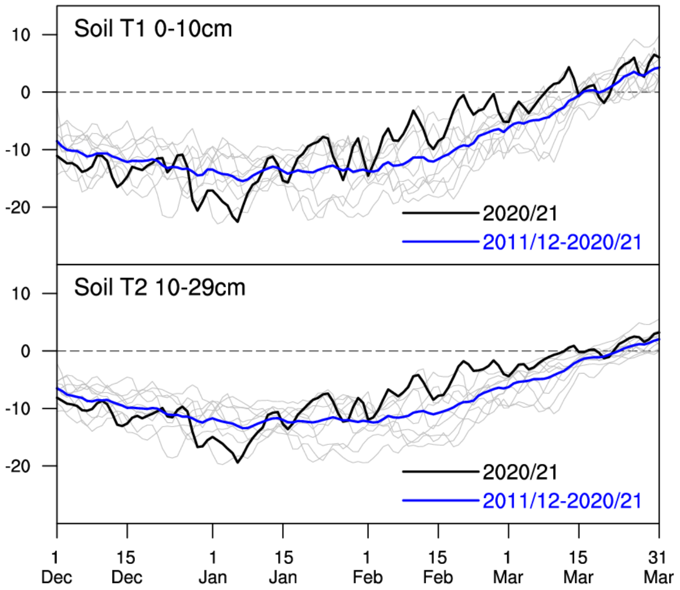

Figure S3. Daily variation in soil temperature (unit: °C, underground depth: 0–10 cm and 10–29cm) from 1 December to 31 March in 2020/21 (black), 2011/12–2020/21 mean (blue) and each year during 2011/12–2019/20 (grey). The data were downloaded from dataset of Global Land Data Assimilation System (i.e., GLDAS, https://ldas.gsfc.nasa.gov/data).

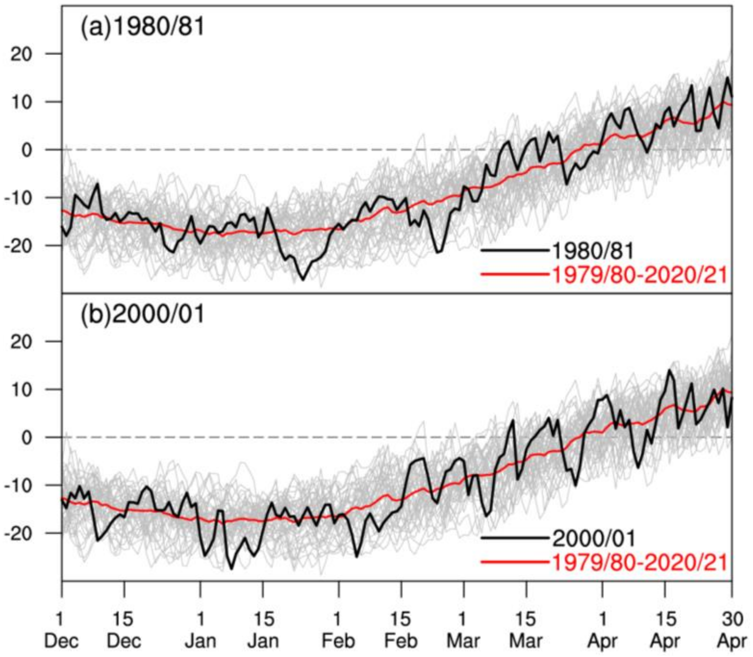

Figure S4. Daily variation in surface air temperature (unit: °C) from (a) 1 December 1980 to 30 April 1981 (black), and (b) 1 December 2000 to 30 April 2001 (black), 1979/80–2020/21 mean (red) and each year during 1979/80–2019/20 (grey).

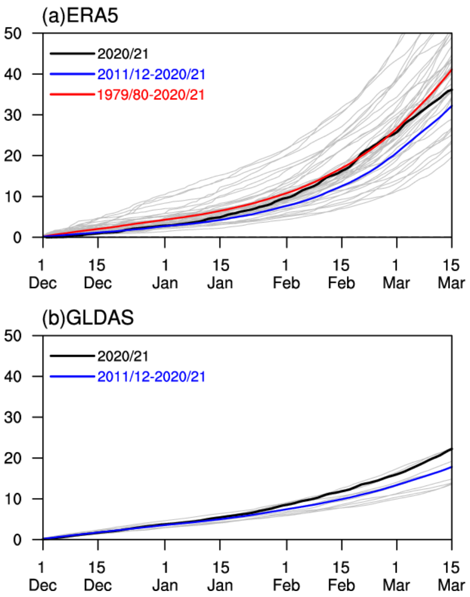

Figure S5. Daily variation in accumulated evaporation in dust source area during 1 December to 15 March in 2020/21 (black), 2011/12-2020/21 mean (blue), 1979/80–2020/21 mean (red) and each year during 1979/80–2019/20 (grey). The results were calculated from (a) ERA5 dataset (unit: 10^{-2} mm) and (b) GLDAS dataset (unit: 10^{-5} kg m^{-2} s^{-1}).

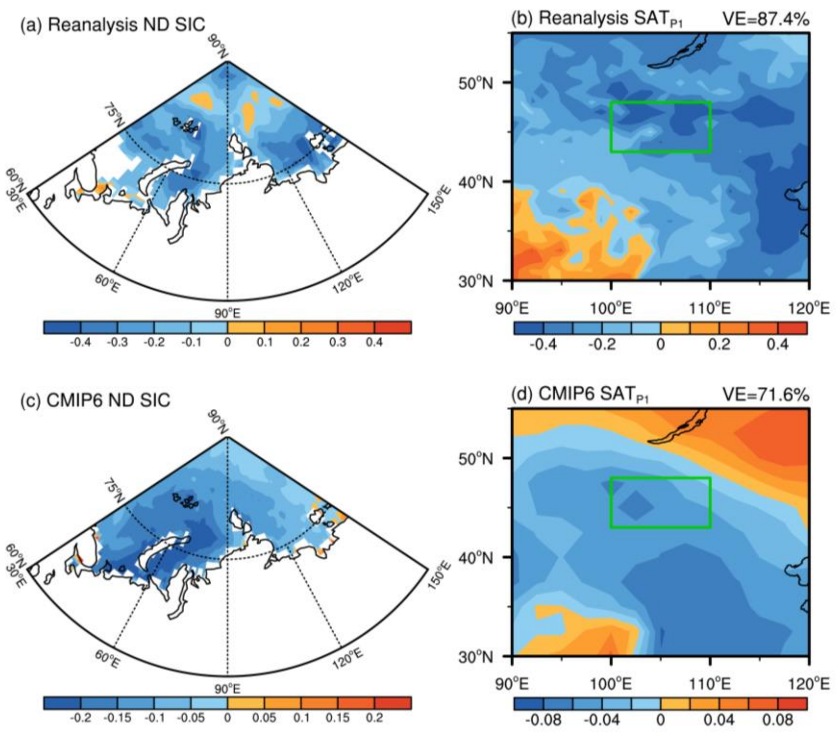

Figure S6. Heterogeneous correlation map of the first SVD mode for detrended (a, c) ND SIC and (b, d) SAT $_{P1}$. The SVD decomposition are based on reanalysis data during 1979/80–2020/21 (a, b) and CMIP6 historical simulations during 1979/80–2013/14 (c, d). The green boxes represent the dust source area. The linear trend are removed before the SVD analysis. The SVD of each CMIP6 model was independently calculated and then the results were averaged as the ensemble mean.

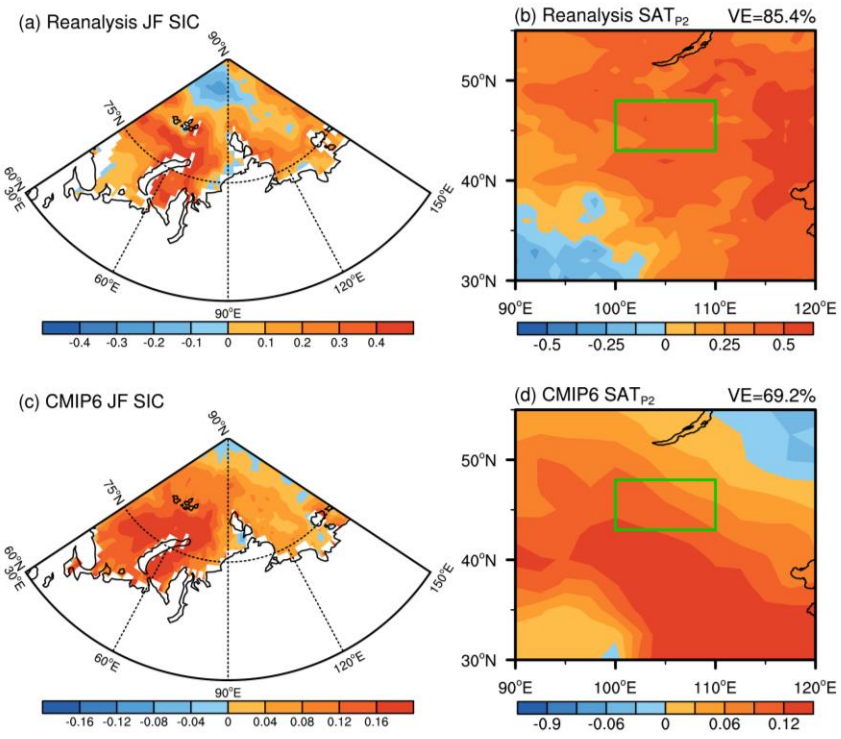

Figure S7. Heterogeneous correlation map of the first SVD mode for detrended (a, c) JF SIC and (b, d) SAT $_{P2}$. The SVD decomposition are based on reanalysis data during 1979/80–2020/21 (a, b) and CMIP6 historical simulations during 1979/80–2013/14 (c, d). The green boxes represent the dust source area. The linear trends are removed before the SVD analysis. The SVD of each CMIP6 model was independently calculated and then the results were averaged as the ensemble mean.

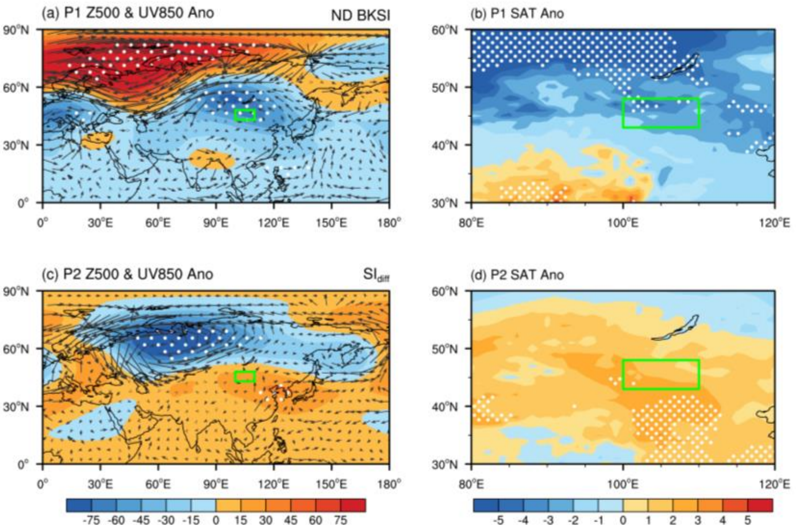

Figure S8. Composite of (a, c) 500hPa geopotential height (unit: gpm, shading) and 850hPa wind (unit: m s^{-1}, vector), and (b, d) SAT (unit: ℃) during P1 (a, b) and P2 (c, d). The composites were carried out between the lowest (less than –1 × standard deviation) and highest (larger than 1 × standard deviation) ND BKSI years (a, b); ND minus JF BKSI years (c, d) from 1979/80 to 2020/21. The white dots indicate that the differences were above the 95% confidence level (*t* test). The green box represents the dust source area. The linear trends are removed.

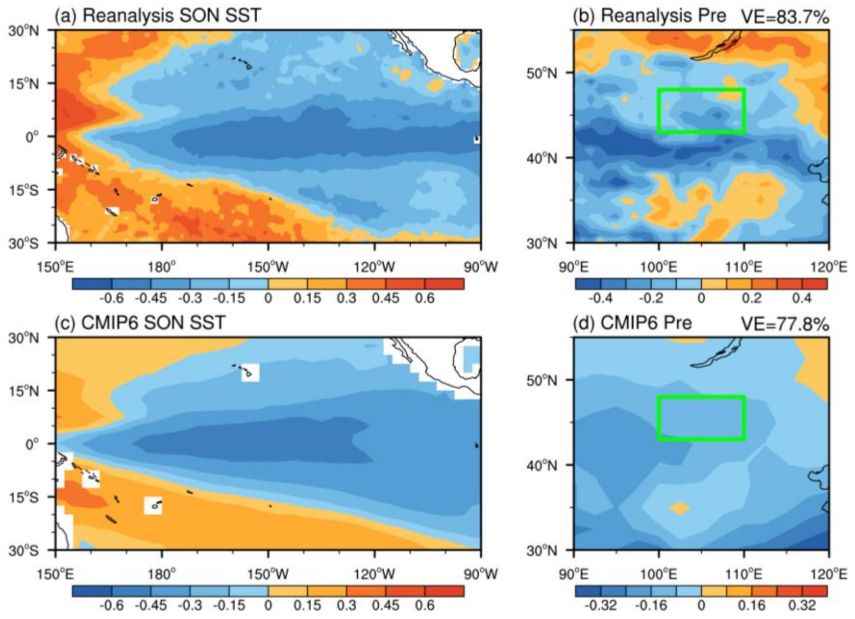

Figure S9. Heterogeneous correlation map of the first SVD mode for detrended (a, c) SON Pacific SST and (b, d) Precipitation during 1 December to 15 March 1979/80–2020/21. The SVD decomposition are based on reanalysis data during 1979/80–2020/21 (a, b) and CMIP6 historical simulations during 1979/80–2013/14 (c, d). The green boxes represent the dust source area. The linear trends are removed before the SVD analysis. The SVD of each CMIP6 model was independently calculated and then the results were averaged as the ensemble mean.

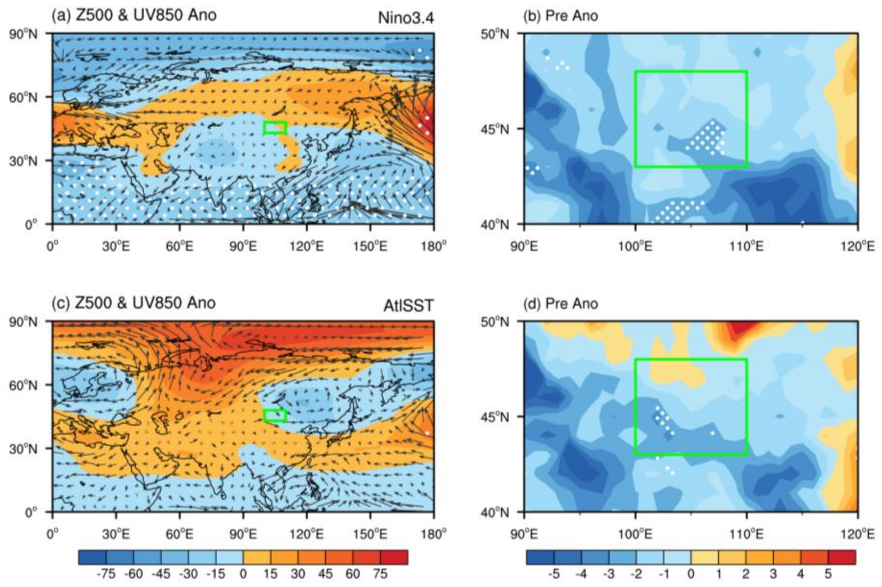

Figure S10. Composite of (a, c) 500hPa geopotential height (unit: gpm, shading) and 850hPa wind (unit: m s^{-1}, vector), and (b, d) precipitation (unit: 10^{-3} mm) between the (a, b) lowest (less than –1 × standard deviation) and highest (larger than 1 × standard deviation) years basing on Nino3.4 and (c, d) and AtlSST index from 1979/80 to 2020/21. The white dots indicate that the differences were above the 95% confidence level (*t* test). The green box represents the dust source area. The linear trends are removed.

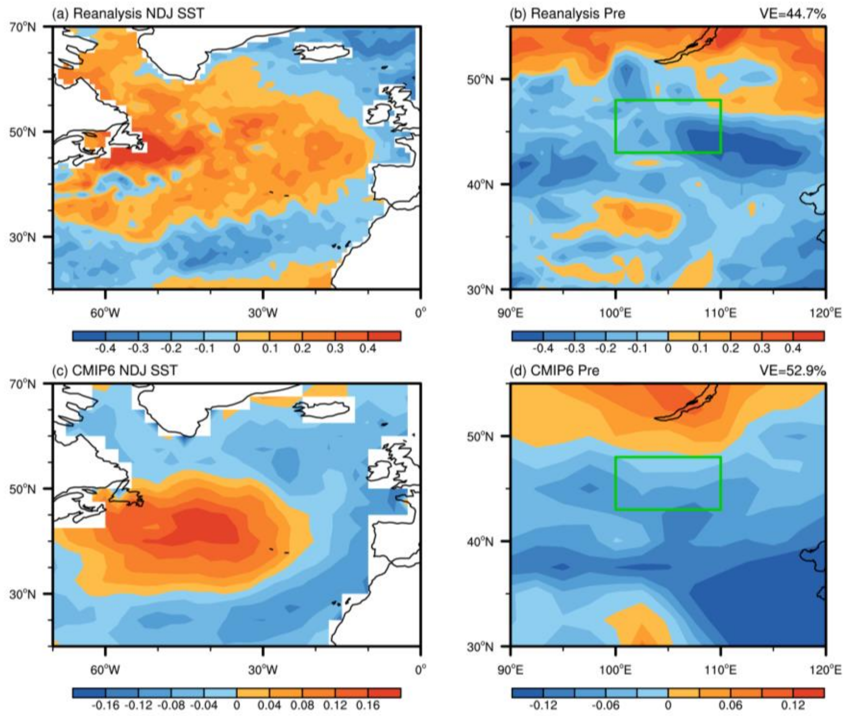

Figure S11. Heterogeneous correlation map of the first SVD mode for detrended (a, c) NDJ Atlantic SST and (b, d) Precipitation during 1 December to 15 March 1979/80–2020/21. The SVD decomposition are based on reanalysis data during 1979/80–2020/21 (a, b) and CMIP6 historical simulations during 1979/80–2013/14 (c, d). The green boxes represent the dust source area. The linear trends are removed before the SVD analysis. The SVD of each CMIP6 model was independently calculated and then the results were averaged as the ensemble mean.

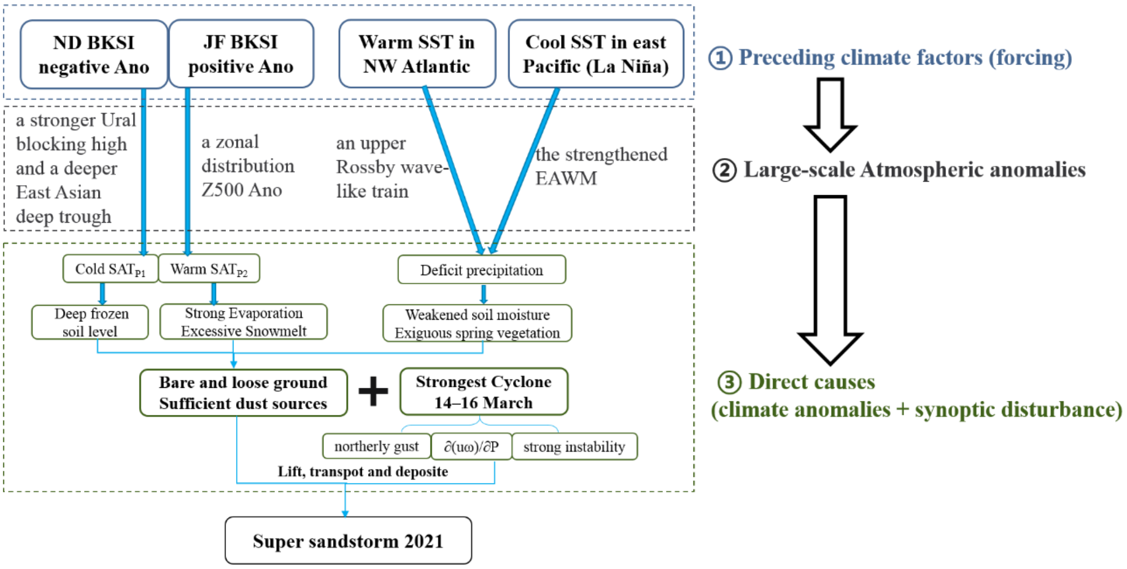

Figure S12. The schematic diagram of how preceding climate factors, large-scale atmospheric anomalies and direct causes impacted super sandstorm 2021 and the linkages among them.

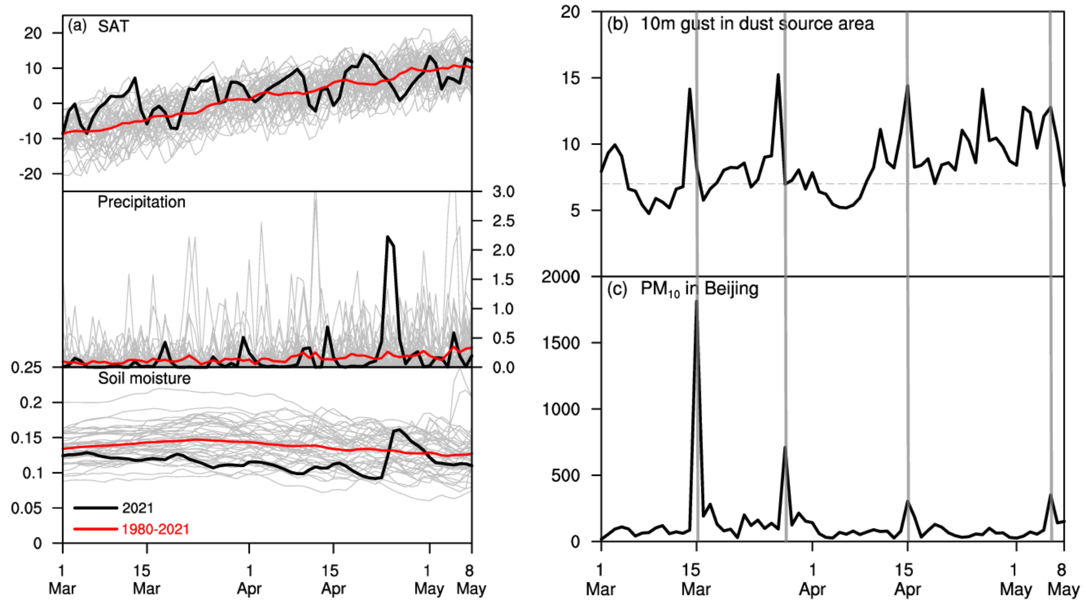

Figure S13. (a) Daily variation in surface air temperature (unit: °C), precipitation (unit: 10^{-2} mm) and soil moisture (unit: 1) in dust source area from 1 March to 8 May 2021 (black), 1980–2021 mean (red) and each year during 1980–2020 (grey). Daily variation in (b)10m gust in dust source area (unit: m s^{-1}) and (c) Beijing PM_{10} concentrations (unit: μg m^{-3}) from 1 March to 8 May 2021 . The grey dotted line in panel (b) represents the gust of 7 m s^{-1}. The grey solid lines represent the processes of dust storm.

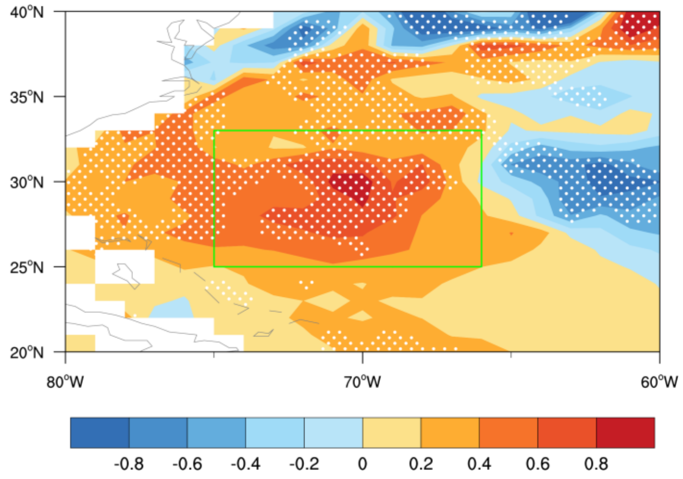

Figure S14. The sea surface temperature anomalies (unit: °C) in September 2020 relative to the mean of 2011-2020. The white dots indicate that the anomalies in 2020 exceeded the 85th percentile of anomalies from 2011 to 2020. The green box represents the key areas influenced the Mongolian cyclone revealed by Wang (2005).
